# Effects of *mscM* Gene on Desiccation Resistance in *Cronobacter sakazakii*

**DOI:** 10.3390/microorganisms12122464

**Published:** 2024-11-30

**Authors:** Dongdong Zhu, Zhengyang Zhang, Ping Li, Xinjun Du

**Affiliations:** State Key Laboratory of Food Nutrition and Safety, College of Food Science and Engineering, Tianjin University of Science and Technology, Tianjin 300457, China; duiduizdd@163.com (D.Z.); zzy2580a@163.com (Z.Z.)

**Keywords:** *Cronobacter sakazakii*, *mscM*, potassium ion, desiccation resistance, biofilm

## Abstract

*Cronobacter sakazakii*, an opportunistic foodborne pathogen, has a strong resistance to osmotic stress and desiccation stress, but the current studies cannot elucidate this resistance mechanism absolutely. A mechanosensitive channel MscM was suspected of involving to desiccation resistance mechanism of *C. sakazakii.* To investigate the specific molecular mechanism, the *mscM* mutant strain (Δ*mscM*) was constructed using the homologous recombination method, and the *cpmscM* complementary strain was obtained by gene complementation, followed by the analysis of the difference between the wild-type (WT), mutant, and complementary strains. Compared to the wild-type bacteria (WT), the inactivation rate of the Δ*mscM* strain decreased by 15.83% (*p* < 0.01) after desiccation stress. The absence of the *mscM* gene led to an increase in the membrane permeability of mutant strains. Through turbidity assay, it was found that the intracellular content of potassium ion (K^+^) of the Δ*mscM* strain increased by 2.2-fold (*p* < 0.05) compared to the WT strain, while other metal ion contents, including sodium ion (Na^+^), calcium ion (Ca^2+^), and magnesium ion (Mg^2+^), decreased by 48.45% (*p* < 0.001), 24.29% (*p* < 0.001), and 26.11% (*p* < 0.0001), respectively. These findings indicate that the MscM channel primarily regulates cell membrane permeability by controlling K^+^ efflux to maintain the homeostasis of intracellular osmotic pressure and affect the desiccation tolerance of bacteria. Additionally, the deletion of the *mscM* gene did not affect bacterial growth and motility but impaired surface hydrophobicity (reduced 20.52% compared to the WT strain, *p* < 0.001), adhesion/invasion capability (reduced 26.03% compared to the WT strain, *p* < 0.001), and biofilm formation ability (reduced 30.19% compared to the WT strain, *p* < 0.05) of the bacteria. This study provides a reference for the role of the *mscM* gene in the desiccation resistance and biofilm formation of *C. sakazakii*.

## 1. Introduction

*C. sakazakii* is a Gram-negative (G^−^) bacterium that can induce deadly bacteremia, meningitis, and necrotizing small intestine colitis and may lead to serious sequelae [1]. Compared to other enterobacteria, this bacterium has strong desiccation resistance and can survive in low-water-activity environments such as infant formula, flour, and cheese powder, thus posing a serious threat to the target population, especially for infants and the elderly with weakened immunity [2]. According to reports, some factors, including unique yellow pigmentation, biofilm components, and osmolytes, are believed to contribute to the desiccation resistance of *C. sakazakii*, but the specific mechanism has not been studied in depth [3,4,5].

Desiccation is a kind of extreme form of osmotic stress, and some related models have been established to explore the desiccation resistance mechanism of *C. sakazakii*. For example, the intracellular accumulation of trehalose or betaine is considered to be an important biological process for restoring osmotic balance and protecting *C. sakazakii* from desiccation stress [6,7]. Additionally, the rapid intracellular accumulation of some electrolytes is believed to assist bacteria in resisting high osmotic pressure [8]. Potassium glutamate and less potassium acetate are believed to provide temporary protection for cells upon hyperosmotic shock, especially the former [9]. When bacteria are dehydrated in a low-water-activity environment, they immediately absorb potassium from the outside and subsequently produce potassium glutamate, which forms the first line of defense to resist hyperosmotic shock. Furthermore, potassium glutamate controls the synthesis and transport of other more effective osmolytes by inhibiting the binding of RNA polymerase and ribosomal promoters [10].

Mechanosensitive (MS) channels located at the cytoplasmic membrane are gated by mechanical forces caused by turgor pressure. Two major types (classification based on conductance) of MS channels have been found in both prokaryotes and eukaryotes, including the mechanosensitive channel of large conductance (MscL) and the mechanosensitive channel of small conductance (MscS) [11,12]. These channels act as “emergency relief valves” that protect cells from lysis during hypoosmotic shock by exporting osmotically active solutes and ions. Additionally, another mechanosensitive channel of miniconductance (MscM) opens through weaker membrane tension. Compared to MscL and MscS, its structure and function is less known [13]. Michelle D [14] believed that MscM is composed of several different channels, including YjeP (also named as MscM protein directly), YbdG, and other possible homologous proteins. The channel composed of YjeP heptamer was considered to be the major component of MscM, and this channel also contributes to resistance against hypoosmotic shock. In short, these non-specific channels that promote the exportation of osmolytes may be crucial for the desiccation resistance of *C. sakazakii*.

In this study, we deleted the homologous *mscM* gene (*ESA_00167*) of *C. sakazakii* and focused on the desiccation resistance of the mutant strains. The deletion of the *mscM* gene led to an increase in intracellular potassium levels and a decrease in bacterial inactivation rate in a dry environment, indicating that MscM negatively regulated the desiccation resistance of *C. sakazakii* by promoting the efflux of K^+^. In addition, the deletion of the *mscM* gene also affected the surface hydrophobicity, adhesion/invasion capability, and biofilm formation of *C. sakazakii*. This study provides a reference for further studying the mechanism of desiccation resistance and other biological characteristics of *C. sakazakii*.

## 2. Materials and Methods

### 2.1. Bacterial Strains, Plasmids, and Growth Conditions

All the strains and plasmids (stored at Tianjin University of Science and Technology) used in this study are shown in Table S1. *C. sakazakii* ATCC BAA-894 (ATCC, Manassas, VA, USA) was the target strain. *Escherichia coli* DH5α (Thermo, Waltham, MA, USA)and DH5α S17-λpir (Thermo, Waltham, MA, USA) were used for gene cloning. All bacterial strains were continuously shaken in a Luria–Bertani (LB) medium at 37 °C for cultivation. The suicide plasmid pCVD442 (Miaolingbio, Wuhan, China) was used for gene editing, and the pACYC184 plasmid (Miaolingbio, Wuhan, China) was used to complement the deletion gene.

### 2.2. Deletion of the mscM Gene and Gene Complementation

The mutant strains were constructed using a reported method [15], with minor modifications. In brief, two pairs of primers (pCVD442-F, pCVD442-R) were used to amplify the pCVD442 plasmid backbone, and the up and down homologous arms of the *mscM* gene were amplified from *C. sakazakii* genome using corresponding primers (*mscM*-UF, *mscM*-UR, *mscM*-DF, and *mscM*-DR). The fragments of the homologous arm were constructed into the linearized pCVD442 plasmids using a ClonExpress^®^ II One Step Cloning Kit (Vazyme, Nanjing, China), and then the recombinant plasmid was transformed into *E. coli* DH5α S17-λpir for storage. A two-step screening method was adopted to perform the *mscM* gene deletion. First, the recombinant plasmids containing the homologous arm fragments were introduced into *C. sakazakii* (electrotransformation), and the screening of the bacterial strains based on the ampicillin resistance was performed. Second, the above strains were inoculated on an LB solid medium containing 30% sucrose to achieve *mscM* gene knockout. As for the Δ*mscM* gene complementation, the amplified *mscM* gene (*cpmscM* F, *cpmscM* R) was constructed into pACYC184 plasmid, followed by transforming into mutant strains to produce the complementary strains harboring the *mscM* gene (*cpmscM* strain). Additionally, the primers including *mscM* 1 F/R, *mscM* 2 F/R, and *mscM* 3 F/R were used to verify the strain mentioned above. The primers used in this study are listed in Table S2.

### 2.3. Bacterial Growth Curves

Bacteria cultured overnight were transferred into fresh LB medium at a ratio of 1:100 for further cultivation. The OD_600nm_ value was measured every 1 h using a Bio-Radometer Plus spectrophotometer (Eppendorf, Hamburg, Germany).

### 2.4. Desiccation Resistance Evaluation

Logarithmic-phase bacteria were inoculated into a 96-well plate (100 μL/well) and further cultured at 37 °C for 24 h. Briefly, 1 μL liquid cultures were continuously diluted in a 50 mM PBS buffer (pH 7.4) and coated on the LB solid medium for counting the initial number of cells. The 96-well plate was then placed in a sterile desiccator and incubated for an additional 9 days at 37 °C. After drying, the residual cells were resuspended in PBS buffer and coated on the LB solid medium for counting. The inactivation rate of bacteria was calculated using the following formula:

Inactivation rate (%) = (A_I_ − A_R_)/A_I_ × 100%

Here, A_I_ represents the initial number of bacteria before drying, and A_R_ represents the residual number of bacteria after drying. The higher inactivation rate indicated the weaker desiccation resistance of bacteria.

### 2.5. Determination of Intracellular K^+^ Contents

Intracellular K^+^ contents were analyzed using a commercial kit. Briefly, bacteria were cultured toOD_600nm_ 0.6 and then collected by centrifugation at 4000× *g* for 5 min at 4 °C. After washing three times, the cells were resuspended in precooled ultrapure water and broken by sonication (300 W, 4 s × 4 s). The sample was further centrifuged at 12,000× *g* for 15 min at 4 °C to isolate the supernatants. The K^+^ contents in the supernatants were measured using a potassium (K) turbidimetric assay kit (Elabscience, Wuhan, China) and calculated using the following formula:

K^+^ contents (mmol/gprot) = (A450 − b)/a × f/Cpr

Here, A450 represents the OD_450nm_ value, a represents the slope of the standard curve, b represents the intercept of the standard curve, f represents the dilution rate, and Cpr represents the protein concentration (gprot/L).

### 2.6. Determination of Total K^+^, Na^+^, Ca^2+^, and Mg^2+^ Contents

The total contents of different ions(including K^+^, Na^+^, Ca^2+^, and Mg^2+^) were determined by atomic absorption spectroscopy. Bacteria were cultured to OD_600nm_ 0.6 and then collected by centrifugation, freeze-dried, and digested with nitric acid in a microwave oven. Subsequently, the total ion contents were carried out using an atomic absorption spectrometer (Thermo, Waltham, MA, USA). The calculation formula is as follows:

Ion (mg/g) = B*d*V/m

Here, B represents the value measured with an atomic absorption spectrometer, d represents the dilution rate, V represents the reaction volume, and m represents the sample weight.

### 2.7. Permeability Assay

1-N-phenylnaphthylamine (NPN) was used to determine the outer membrane permeability. Logarithmic-phase bacteria were washed three times and resuspended in a PBS buffer, and the OD_600nm_ values were adjusted to 0.5. Subsequently, NPN was added to the bacterial suspension at the final concentration of 40 μΜ in the darkness, and the fluorescence value was measured using a microplate reader (Sunrise-Basic, Vienna, Austria) with excitation and emission wavelength of 350 nm and 420 nm, respectively.

### 2.8. Bacterial Adhesion/Invasion

HCT-8 cells (ATCC, Manassas, VA, USA) were used to test the bacterial adhesion/invasion ability. Briefly, HCT-8 cells were cultured in RPMI 1640 medium (Gibco, Stockrick, CA, USA) containing a 10% fetal bovine serum (Gibco, Stockrick, CA, USA) until monolayer cells covered the bottom of a 24-well plate. After washing three times with the PBS buffer, the logarithmic-phase bacteria were resuspended in an RPMI 1640 medium (10% fetal bovine serum). Bacteria were added to the 24-well plate in an equal amount (about 0.5 × 10^7^ CFU/well) and incubated for 3 h. Subsequently, the HCT-8 cells were washed three times with the PBS buffer to remove unattached bacteria and further lysed with 0.1% Triton-100. Finally, the lysate was diluted with PBS, coated on the LB solid medium, and incubated at 37 °C overnight for the adhesion/invasion rate assay.

### 2.9. Hydrophobicity Assay

The surface hydrophobicity of bacteria was determined with xylene. Logarithmic-phase bacteria were washed three times with the PBS buffer, and the OD_600nm_ values were adjusted to 0.5. Then, 2 mL of bacteria suspension was mixed with 800 μL of xylene and placed at room temperature for 3 h. After removing the upper organic phase, the absorbance of the aqueous phase was measured at OD_600nm_. The formula was calculated as follows:

Hydrophobicity (%) = (0.5 − A600)/0.5 × 100%.

where 0.5 represents the initial absorbance at OD_600nm_, and A600 represents the absorbance of the aqueous phase.

### 2.10. Biofilm Formation

The biofilm formation was determined by crystal violet (CV) staining. Bacteria were cultured to OD_600nm_ 0.6 and then transferred to a 96-well plate (LB medium, 100 μL/well) and further cultured at 37 °C for 48 h to establish a biofilm. The biofilm was fixed with 99% methanol and stained with 0.1% CV (Solarbio, Beijing, China) for 30 min. Then, 95% ethanol was used for decolorization. The absorbance was detected with a microplate reader (Sunrise-Basic, Austria) at OD_570nm_.

### 2.11. Statistical Analysis

Statistical analysis was carried out with Prism 8.0 software. The significant differences in the results were assessed using Duncan’s test or analysis of variance (ANOVA). A threshold below a *p*-value of 0.05 was considered statistically significant (* *p* < 0.05, ** *p* < 0.01, *** *p* < 0.001, and **** *p* < 0.0001). Each experiment was repeated independently at least three times, and the results were expressed as mean ± deviation.

## 3. Results

### 3.1. Construction of the mscM Mutant Strain

**Figure 1:**
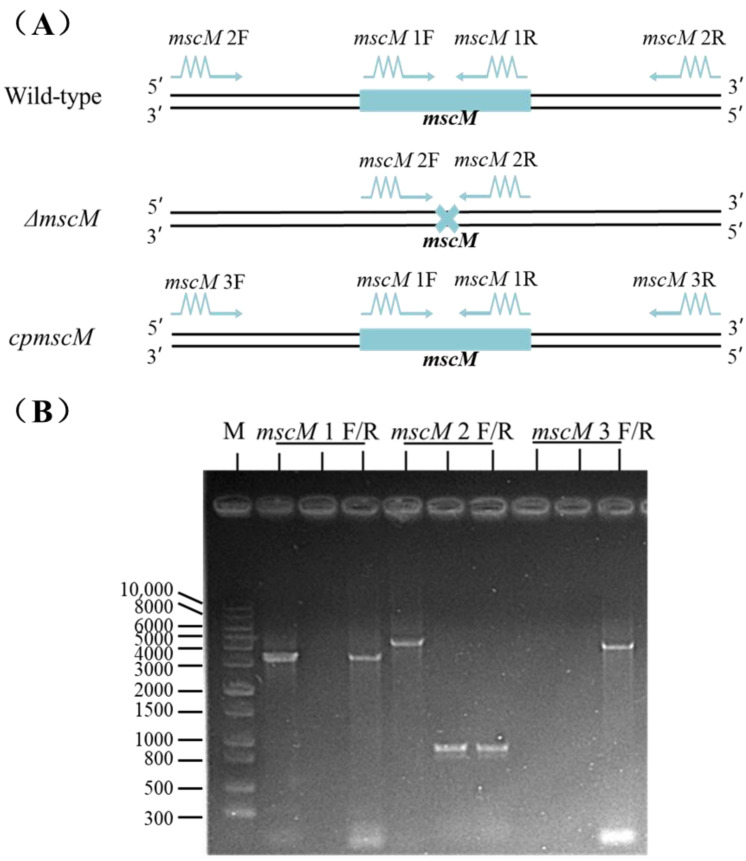
Locations of specific primers and PCR amplification: (**A**) Locations of specific primers used for PCR validation; (**B**) PCR amplification result from different bacterial strains using different primers pairs, M. Maker; *msccM* 1 F/R, the WT strain; *msccM* 2 F/R, the Δ*mscM* strain; *msccM* 3 F/R, the *cpmscM* strain.

The suicide plasmid pCVD442 was used to knock out the *mscM* gene of *C. sakazakii*. The amplification location of verified primers in the different *C. sakazakii* strains is shown in Figure 1A. When using *mscM* 1 F/R as primers, a 3378 bp band was amplified from the WT and *cpmscM* strains, and no band was amplified from the Δ*mscM* strain (Figure 1B). An expected band (4334 bp) was obtained from the WT strain, while a shorter band (1001 bp) was amplified from the Δ*mscM* or *cpmscM* strains when using the *mscM* 2 F/R primer. In addition, the *mscM* 3 F/R primer could amplify an expected band (4020 bp) on the pACYC184 plasmid in the *cpmscM* strains (Figure 1B). These results indicated that the *mscM* gene was deleted in the Δ*mscM* strain, and this gene was also complemented in the *cpmscM* strain.

### 3.2. Effects of MscM on the Bacterial Growth Curves

**Figure 2:**
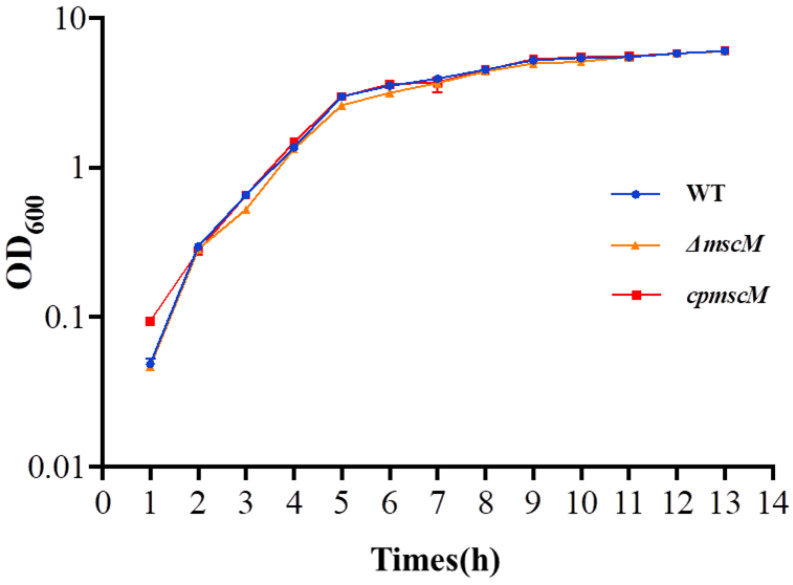
The growth curves of WT, Δ*mscM*, and *cpmscM* strains.

To determine whether the *mscM* gene was essential for *C. sakazakii*, the growth curves of the three groups of bacteria (WT, Δ*mscM*, and *cpmscM*) were recorded. As shown in Figure 2, the WT, Δ*mscM*, and *cpmscM* strains shared similar growth trends, indicating that the *mscM* gene is a non-lethal gene, and its absence does not impair the bacterial vitality and growth, thus ruling out the possible growth differences that would interfere with subsequent experiments.

### 3.3. Effects of MscM on the Desiccation Resistance of C. sakazakii

**Figure 3:**
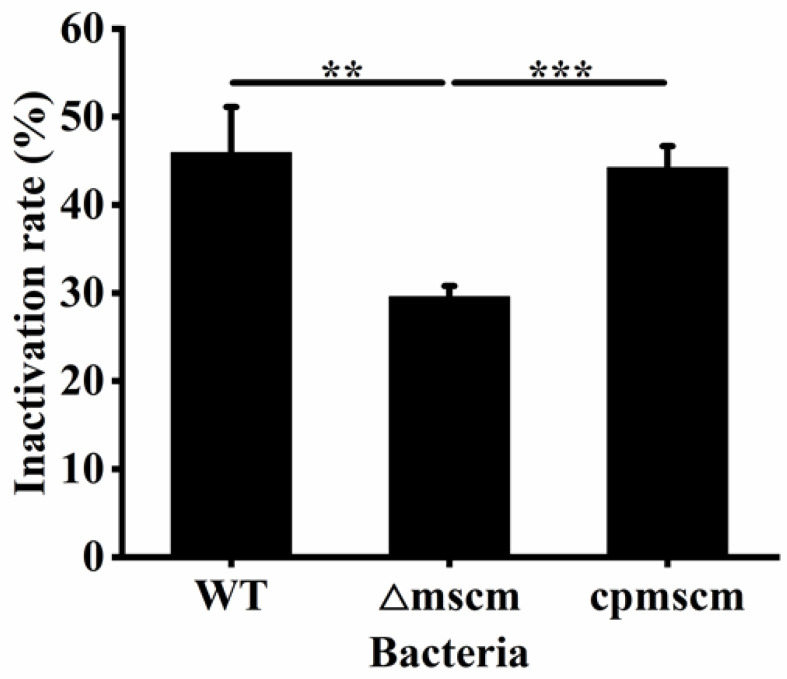
The drying inactivation rate of WT, Δ*mscM*, and *cpmscM* strains. ** *p* < 0.01, *** *p* < 0.001.

To investigate the effects of the MscM channel on the desiccation resistance of *C. sakazakii*, the desiccation resistance ability of the three strains, namely WT, Δ*mscM*, and *cpmscM,* was evaluated. As shown in Figure 3, the inactivation rate of the WT strain was 45.94 ± 5.18% after 9 days of drying treatment, while the inactivation rate of the Δ*mscM* strain was 30.11 ± 1.47%. The inactivation rate of the Δ*mscM* strain was reduced by 15.83% (*p* < 0.01) compared with the WT strain. In addition, the inactivation rate of the *cpmscM* strain was 44.26 ± 2.43%, which was not significantly different from that of the WT strain. Therefore, the MscM protein had a negative effect of the regulation on the desiccation resistance of *C. sakazakii*.

### 3.4. The Different Ion Contents in WT, ΔmscM and cpmscM Strains

**Figure 4:**
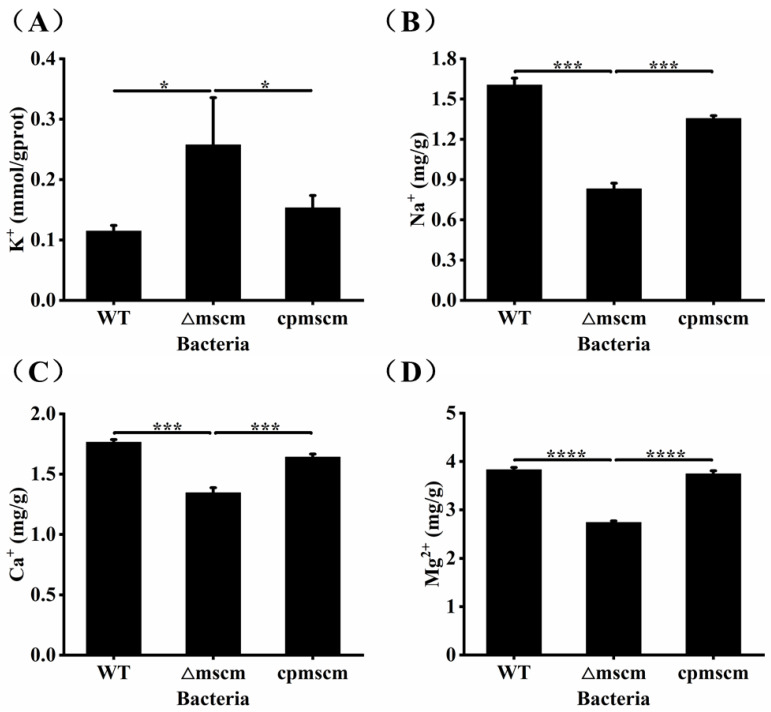
The different ion contents in WT, Δ*mscM*, and *cpmscM* strains: (**A**) intracellular potassium ion concentration; (**B**–**D**) total Na^+^, Ca^2+^, and Mg^2+^ ion contents. * *p* < 0.05, *** *p* < 0.001, **** *p* < 0.0001.

As an ion channel, the reported MS channel has the ability to maintain the osmotic balance of the cells by regulating the efflux of metal ions. In order to study the molecular mechanism of MscM related to the desiccation resistance of *C. sakazakii*, the levels of intracellular potassium were detected. As shown in Figure 4A, the intracellular potassium ion concentration of the WT strain was 0.11 ± 0.01 mM/gprot, which was significantly higher than that of the Δ*mscM* strain, at 0.26 ± 0.08 mM/gprot (*p* < 0.05). As for the *cpmscM* strain, the complementary *mscM* gene promoted a decrease in cytoplasmic K^+^ to 0.15 ± 0.02 mM/gprot. In addition, the total K^+^ content of the WT, Δ*mscM*, and *cpmscM* strains were 5.89 ± 0.14 mg/g, 6.80 ± 0.08 mg/g, and 5.94 ± 0.18 mg/g, respectively (Figure S1). Consistent with the changing trend of intracellular potassium ion concentration, the total K^+^ content of the Δ*mscM* strain also increased compared to the WT strain. Other metal ions also contribute to the osmotic balance. To further explore the effects of the *mscM* gene on the other metal ion contents, we detected the total ion contents of Na^+^, Ca^2+^, and Mg^2+^ ions among the three strains. As shown in Figure 4B–D, the total ion contents of Na^+^, Ca^2+^, and Mg^2+^ were 0.83 ± 0.04 mg/g, 1.34 ± 0.04 mg/g, and 2.83 ± 0.03 mg/g in the Δ*mscM* strain, which were less than the values in the WT strain 1.61 ± 0.05 mg/g (*p* < 0.001), 1.77 ± 0.02 mg/g (*p* < 0.001), and 3.83 ± 0.04 mg/g (*p* < 0.0001), respectively. The total contents of the three ions were 1.35 ± 0.02 mg/g (Na^+^), 1.64 ± 0.03 mg/g (Ca^2+^), and 3.74 ± 0.06 mg/g (Mg^2+^) in the *cpmscM* strain, indicating a successful gene complementation. The above results indicate that *C. sakazakii* can control the K^+^ efflux through MscM channels, and the deletion of the *mscM* gene may stimulate the opening of other MS channels, thereby promoting the efflux of Na^+^, Ca^2+^, and Mg^2+^.

### 3.5. Detection of the Outer Membrane Permeability

**Figure 5:**
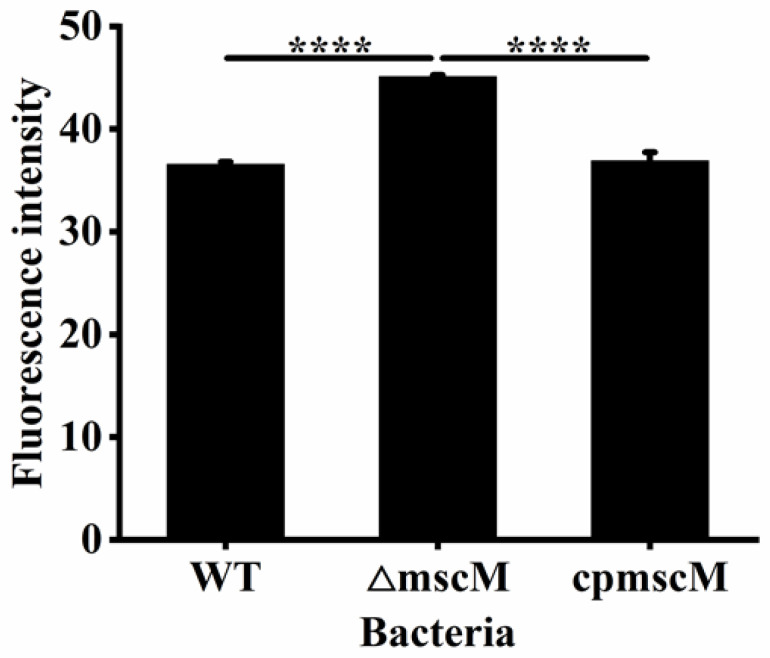
Outer membrane permeability. **** *p* < 0.0001.

The outer membrane permeability is an essential indicator for G^−^ bacteria to respond to osmotic stress and regulate osmotic balance. To investigate the effects of the MscM channel on the outer membrane permeability, NPN was used as the fluorescent probe in this study. As shown in Figure 5, the fluorescent value of the Δ*mscM* strain was 45.09 ± 0.17, which was about 19.05 ± 0.006% (*p* < 0.0001) and 18.19 ± 0.02% (*p* < 0.0001) higher than the WT (36.5 ± 0.24) and *cpmscM* (36.89 ± 0.84) strains, respectively, indicating that the outer membrane permeability was increased in the Δ*mscM* strain, and the MscM channel had a negative regulatory effect on it.

### 3.6. Effects of MscM on Bacterial Adhesion/Invasion

**Figure 6:**
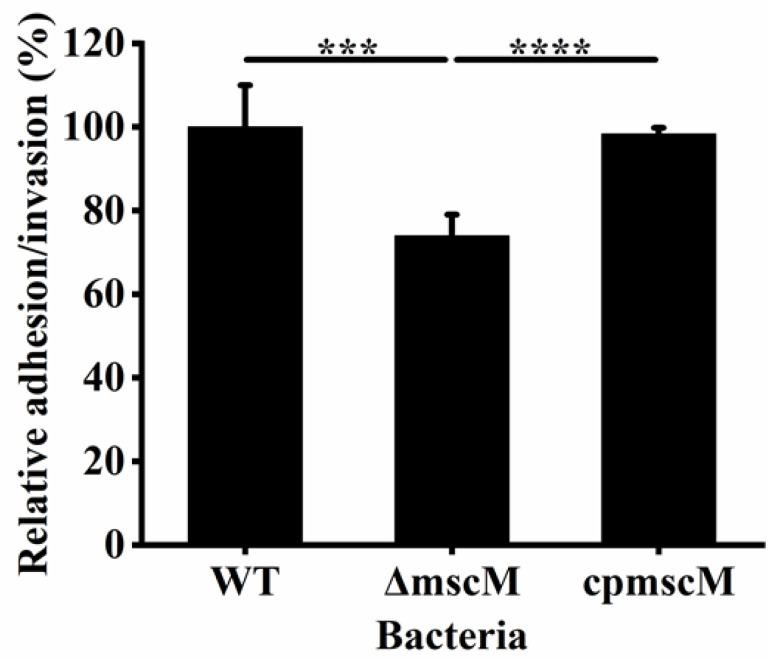
Relative adhesion/infestation rate of the different strains.*** *p* < 0.001, **** *p* < 0.0001.

Bacterial attachment is related not only to pathogenicity but also to growth, aggregation, and colonization. To study the effects of MscM protein on bacterial attachment, HCT-8 cells were used to determine the adhesion/invasion of the different strains. As shown in Figure 6, compared to the WT and *cpmscM* strains, the adhesive/invasive rate decreased to 73.97 ± 5.07% in the Δ*mscM* strain, indicating that the deletion of the *mscM* gene damaged the adhesion/invasion of *C. sakazakii*.

### 3.7. Effects of MscM on the Surface Hydrophobicity

**Figure 7:**
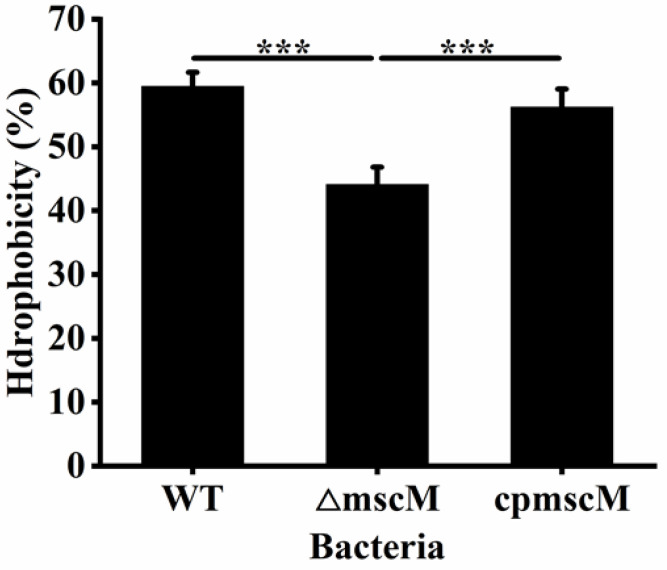
The surface hydrophobicity of bacteria. *** *p* < 0.001.

The surface hydrophobicity is vital for bacterial adhesion and aggregation. To study the surface hydrophobicity of the three strains (WT, Δ*mscM*, and *cpmscM*), the bacteria were treated with xylene and the absorbance of the aqueous phase was recorded. As shown in Figure 7, the surface hydrophobicity values were 59.47 ± 2.81%, 44.09 ± 2.74%, and 56.22 ± 2.83% in the WT, Δ*mscM*, and *cpmscM* strains, respectively. The surface hydrophobicity of the Δ*mscM* strain was 20.52% (*p* < 0.001) lower than that of the WT strain, indicating that the absence of the *mscM* gene had an adverse effect on bacterial surface hydrophobicity.

### 3.8. Effects of MscM on the Biofilm Formation

**Figure 8:**
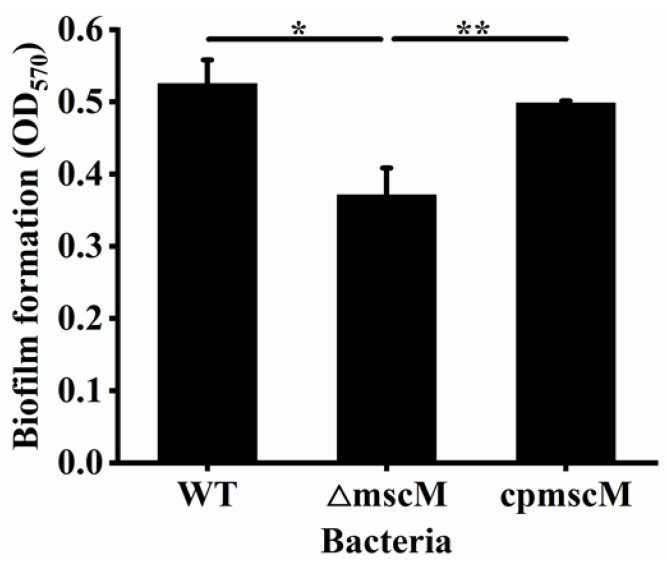
Biofilm formation. * *p* < 0.05, ** *p* < 0.01.

Biofilms have been shown to be associated with most bacteria overcoming environmental barriers and surviving for a long time. To investigate the effect of the MscM channel on biofilm formation, the biofilm formation of the three strains was detected by CV staining. The results are illustrated in Figure 8. The OD_570nm_ value of the WT strain was 0.53 ± 0.04, while that of the Δ*mscM* strain was 0.37 ± 0.03. The biofilm formation of the Δ*mscM* strain decreased by 30.19% (*p* < 0.05) when lacking MscM protein, whereas the complementary MscM protein could restore the biofilm formation to the normal level (0.49 ± 0.003), indicating that the presence of the MscM channel was beneficial for the biofilm formation of *C. sakazakii*.

## 4. Discussion

**Figure 9:**
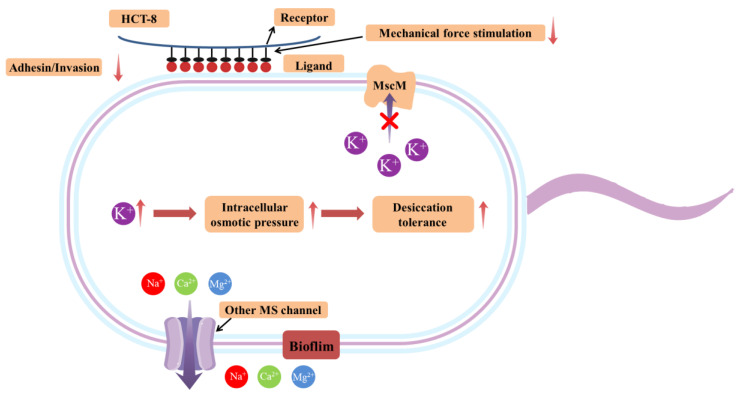
The illustration of the effects of MscM proteins on *C. sakazakii*.

As an opportunistic pathogen, *C. sakazakii* is famous for its desiccation resistance. Several models have been established to clarify the molecular mechanism of desiccation resistance of this bacterium, but the special mechanism still harbors numerous ambiguities. In this study, the deletion of the *mscM* gene was used to research the effect of this gene on the desiccation resistance of *C. sakazakii*. In addition, the changes in some biological characters caused by the absence of the *mscM* gene were also explored in *C. sakazakii*. The physiological effect of MscM proteins on *C. sakazakii* is shown in Figure 9.

The deletion of the *mscM* gene did not impair bacterial vitality and growth and enhanced the desiccation resistance of *C. sakazakii*. The opening of MS channels was crucial for bacterial survival when osmolarity changed. For example, it has been found that the MscS and MscL channels contributed to maintaining the integrity of the bacterial envelope during osmotic shock [16]. Solutes flow out through these channels to reduce the water influx rate and avoid cell lysis. In our study, compared with the WT strain, the Δ*mscM* strain accumulated more intracellular K^+^ and total K^+^ contents, indicating that the MscM channel primarily downregulated the desiccation resistance of *C. sakazakii* by reducing the efflux of K^+^. The phenomenon that the potassium efflux damaged the capacity of bacterial desiccation resistance was also reported in other studies [17,18]. The high concentration of potassium glutamate was considered to compose the first line of defense to resist the desiccation. However, it was also a less preferred osmolyte due to its ability to disrupt cellular metabolism [9,19]. The accumulation of potassium glutamate was accompanied by the production of other theoretical osmoprotectants. These osmoprotectants might form the second line of defense in resisting the desiccation and did not induce disturbances to cellular metabolism [20,21]. In this study, the total contents of Na^+^, Ca^2+^, and Mg^2+^ decreased in the Δ*mscM* strain, leading to the dilution of intracellular ion concentrations that promote the water absorption of *C. sakazakii*. This manner might reduce the effective concentration of K^+^ to a certain extent and minimize the damage to cells. These ions might export through other MS channels, but we did not detect any changes in related gene expression by using RT-qPCR (Figure S2). In fact, the opening of MS channels was unable to be detected in the body. Theoretically, they would successively open as the membrane tension increased [13]. In addition, the absence of the *mscM* gene also increased the membrane permeability of the mutant strains. Additionally, the defective membrane could lead to the leakage of other intracellular molecules, such as DNA and proteins, which would cause an adverse effect on bacterial survival in dry environments [17,22]. Bacteria could adapt to extremely dry environments by regulating the expression of the genes related to the desiccation resistance of *C. sakazakii*. The transcription level of the *rpoS* gene was increased, while that of the *betI* gene was decreased in the Δ*mscM* strain (Figure S2). RpoS is the main signal to respond to the osmotic pressure, and BetI was involved in the inhibition of the biosynthesis of the osmoprotectant glycine betaine [3,23]. Both changes in the above two genes helped the bacteria to cope with the desiccation stress. In short, several direct or indirect cascades of events contribute to the desiccation adaption of bacteria and lead to cellular survival.

Biofilm has been shown to be involved in the survival and infection of *C. sakazakii*, and the adhesion and colonization of bacteria are crucial for the formation of their biofilms [24,25]. Compared to the WT strain, the Δ*mscM* strain had defects in biofilm formation and poorer adhesion to HCT-8 cells. The above differences were indeed caused by the lack of MscM protein in the Δ*mscM* strain rather than the differences in bacterial vitality, as the growth curves of the Δ*mscM* and WT strains were almost identical. MS channels could stimulate the expression of the related proteins located in the cell membrane and could improve bacterial adhesion and biofilm formation [26]. In *Pseudomonas aeruginosa*, stimulating MS channels could promote cell growth and biofilm formation [27]. The deletion of the *mscM* gene also impaired the surface hydrophobicity of the Δ*mscM* strain. Surface hydrophobicity is based on the interactions of different molecules in the cell membrane and plays an important role in cell adhesion. When a hydrophobic amino acid was replaced in the transmembrane domain of the MscL protein, it reduced the synthesis of the MscL protein and damaged the surface hydrophobicity of *E*. *coli* [28]. These complicating factors ultimately led to failure in biofilm formation. The poor biofilm formation may provide little assistance for the environmental persistence of bacteria, especially in a dry environment. However, the inactivation rate of the Δ*mscM* strain was lower than the WT strain after a short-term drying treatment (drying for 9 days). *C. sakazakii* could survive in a low-water-activity environment for 1–2 years [29]. We might detect a higher inactivation rate caused by poor biofilm formation in the mutant strains after a longer drying process. Therefore, from another perspective, this also reflects that the accumulation of potassium is crucial for the initial survival of *C. sakazakii* in a low-water-activity environment.

The absence of the *mscM* gene did not affect bacterial motility, but the weakening of adhesion ability also indicated that the MscM protein might play an important role in the pathogenicity of *C. sakazakii*. Bacterial adhesion is key to causing infection. It has been reported that the MS channel protein YnaI of *Salmonella* is essential for intestinal colonization, and the deletion of the *ynaI* gene impairs the adhesion of *S*. *typhimurium* [30]. In addition, the mechanical forces produced in the adhesion process could pull the bacterial envelope, thus causing conformational changes in MS proteins and activating the MS channel, which was beneficial for the interaction between bacterial integrins (the major adhesive mediators of bacteria) and the host cell membrane and generating adhesion signals [31]. Of course, the effects of the MscM protein on the pathogenicity of *C. sakazakii* need further investigation.

## 5. Conclusions

In this study, we mainly reported the effects of the MscM protein on the desiccation resistance of *C. sakazakii*. The deletion of the *mscM* gene could reduce K^+^ efflux and improve the desiccation resistance of *C. sakazakii*, indicating that the intracellular accumulation of K^+^ was important for the tolerance of this bacterium in a dry environment. Additionally, the *mscM* gene mutant strains showed failure in biofilm formation and bacterial adhesion/invasion. This study enhances our understanding of the function of the *mscM* gene and the desiccation resistance mechanism of *C. sakazakii*.

## Data Availability

The original contributions presented in this study are included in the article/Supplementary Material. Further inquiries can be directed to the corresponding authors.

## Supplementary Materials

The following supporting information can be downloaded at: https://www.mdpi.com/article/10.3390/microorganisms12122464/s1, Table S1. Bacterial strains and plasmids used in this study. Table S2. Primers used in this study. Figure S1. The total K^+^ contents in WT, Δ*mscM* and *cpmscM* strains. Figure S2. The transcription level of some genes in Δ*mscM* strains. Figure S3. The bacterial motility of WT, Δ*mscM* and *cpmscM* strains.
