# Supplementary material for: Effects of mscM Gene on Desiccation Resistance in Cronobacter sakazakii
Source: Microorganisms. 2024 Nov 30;12(12):2464. doi: 10.3390/microorganisms12122464 (PMC11678165; doi:10.3390/microorganisms12122464)
Supplement: Supplementary file 1 [file microorganisms-12-02464-s001.zip › microorganisms-3325064-supplementary.pdf]

Supporting Information

Effects of *mscM* gene on desiccation resistance in *Cronobacter sakazakii*

Dongdong Zhu †, Zhengyang Zhang †, Ping Li * and Xinjun Du *

State Key Laboratory of Food Nutrition and Safety, College of Food Science and Engineering,
Tianjin University of Science and Technology, Tianjin 300457, China;
duiduizdd@163.com (D.Z.); zzy2580a@163.com (Z.Z.)

* Correspondence: author: zoelxx@tust.edu.cn (P.L.); xjdu@tust.edu.cn (X.D.);
Tel./Fax: +86-22-60912484 (P.L. & X.D.)

† These authors contributed equally to this work.

SI Materials and Methods

1.1 The expression of other related genes

The expression levels of several genes were monitored in WT, *ΔmscM* and *cpmscM* strains. Briefly, PrimeScript™ II Reverse Transcriptase kit (Takara, Kyoto, Japan) was used to construct cDNA after extracting the total RNA. 16S rRNA was used as internal reference, and then real-time quantitative PCR (RT-qPCR) was carried to detect the transcription levels of the relative genes. The transcription level of the corresponding genes was analyzed using the $2^{-\Delta\Delta Ct}$ method. The related primers were listed in **Table S1**.

1.2 Bacterial motility

The bacterial motility was observed on LB semi-solid medium. Briefly, bacteria were cultured to OD_{600nm} 0.6, and then different groups of bacteria (WT, *ΔmscM* and *cpmscM*) were added to LB medium in an equal amount (0.3% agar powder) and incubated at 30°C for 16 hours. Photographs were taken to record the movement of the bacterial colony.

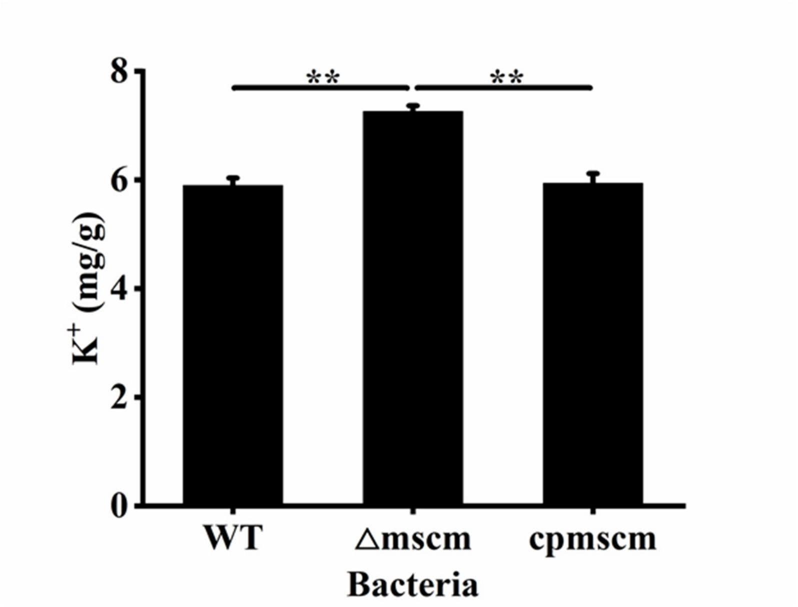

Figure S1. The total K^+ contents in WT, *ΔmscM* and *cpmscM* strains.

2.1 RT-qPCR analysis of the expression of the related genes

The quantities of gene transcription were analyzed by RT-qPCR, and the result was described in **Figure S2**. The transcription levels of Esa_00166 (upstream gene of *mscM*) and Esa_00168 (downstream gene of *mscM*) were 100% and 100% of *ΔmscM* strains compared to WT strains, respectively, indicating that deletion of *mscM* gene did not damage the expression of the genes on both sides. There was also no significant difference in the expressions of other MS channel proteins such as YbiO, MscS, MscK and MscL among WT, *ΔmscM* and *cpmscM* strains. However, the transcription levels of *rpoS* and *betI* that were related to the drought resistance of bacteria increased by 31.59% ± 7.26% and 47.43% ± 5.88% in *ΔmscM* strains, respectively.

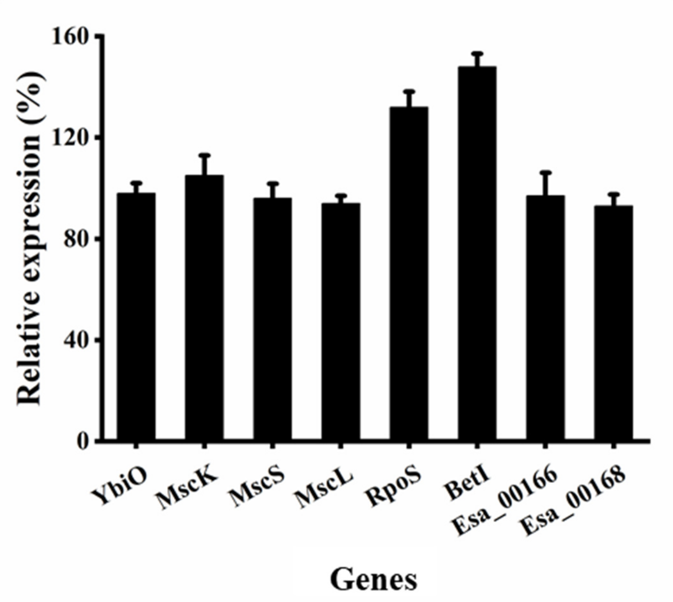

Figure S2. The transcription level of some genes in*ΔmscM* strains.

2.2 Effects of the MscM on the bacterial motility

Bacterial motility was analyzed through the movement rings on LB semi-solid medium. **Figure S3** showed that the three groups of bacteria including WT, *ΔmscM* and *cpmscM* strains formed movement rings of analogous sizes on the medium, which indicated that their hold the same motility and the *mscM* gene had no effect on the motility of *C. sakazakii*.

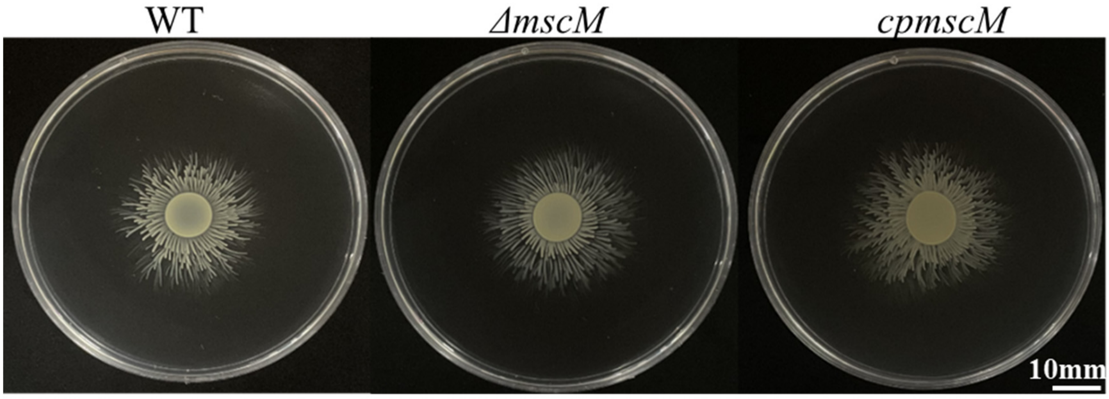

Figure S3. The bacterial motility of WT, *ΔmscM* and *cpmscM* strains.

Table S1 Bacterial strains and plasmids used in this study.

Strain or plasmid	characteristics	Resistance
Cronobacter sakazakii ATCC BAA-894	Wild strains	No
ΔmscM	Mutant strins	No
cpmscM	Δ*mscM* with pACYC184-*mscM*	chloramphenicol
E. coli DH5α	Host bacteria	No
E. coli S17 lambda pir	Host bacteria	No
pCVD442	Suicide plasmid	Ampicillin
pACYC184	Low-copy plasmid	chloramphenicol
pACYC184-*mscM*	pACYC184 with *mscM* gene	chloramphenicol

Table S2 Primers used in this study.

Name	Primer sequences
pCVD442-F	CAATAACCCTGATAAATGCTTCAA
pCVD442-R	CTCATGAGCGGATACATATTTG
mscM U-F	CAATAACCCTGATAAATGCTTCAACAAGGTCAACATGAGCGAGGCG
mscM U-R	GAACGGCATCTCGATGCAGCACGTAAAGGATCAGGCG
mscM D-F	TCCTTTACGTGCTGCATCGAGATGCCGTTCCCGCCGT
mscM D-F	CTCATGAGCGGATACATATTTGTACCGCCTGATGAACGAGGTGG
cpmscM F	CCCAAGCTTGCGGGCAATCTCACGGAAGC
cpmscM R	CGCGGATCCCGACCAGGACTGACCAGCATC
mscM1-F	GATCCTTTACGTGCTGCTGG
mscM1-R	GCGTCACCTCTTCGCTGTTA
mscM2-F	GGCAAAGTGGAGCTGGTT
mscM2-R	TCACGATGCTGGAGTGGTAT
mscM3-F	AAGGCTAAACTGAAAGGACAAG
mscM3-R	CACGGAAACCGAAGACCA
